# Effect of lumbar spinal manipulation on local and remote pressure pain threshold and pinprick sensitivity in asymptomatic individuals: a randomised trial

**DOI:** 10.1186/s12998-016-0128-5

**Published:** 2016-12-05

**Authors:** Sasha L. Dorron, Barrett E. Losco, Peter D. Drummond, Bruce F. Walker

**Affiliations:** 1 Discipline of Chiropractic, School of Health Professions, Murdoch University, 90 South Street, Murdoch, WA 6155 Australia; 2 Discipline of Psychology, School of Psychology and Exercise Science, Murdoch University, 90 South Street, Murdoch, WA 6155 Australia

**Keywords:** Spinal manipulative therapy, Lumbar spine, Pain sensitivity, Pressure pain threshold, Pinprick sensitivity

## Abstract

**Background:**

The mechanisms of clinical pain relief associated with spinal manipulative therapy (SMT) are poorly understood. Our objective was to determine whether lumbar high-velocity low-amplitude SMT altered pressure pain threshold (PPT) and pinprick sensitivity (PPS) locally and remotely, how long any change lasted (up to 30 min), and whether changes related to the side of SMT.

**Methods:**

Thirty-four asymptomatic participants (mean age 22.6 years ±4.0) received a right- or left-sided lumbar SMT. PPT and PPS were measured bilaterally at the calf, lumbar spine, scapula, and forehead before and immediately, 10, 20, and 30 min after intervention. Data were collected between October 2014 and June 2015.

**Results:**

Bilateral calf and lumbar spine PPT increased significantly after 10 – 20 min and was maintained at 30 min (7.2–11.8 % increase). PPS decreased significantly in all locations at various times (9.8 – 22.5 % decrease). At the calf and lumbar spine, PPT increased slightly more ipsilateral to the SMT than contralateral.

**Conclusions:**

Lumbar SMT reduced deep pressure sensitivity locally and in the lower limbs for at least 30 min, whereas sensitivity to pinprick was reduced systemically. These findings suggest that SMT specifically inhibits deep pressure sensitivity distally. These findings are novel compared to other lumbar SMT studies, and may reflect a local spinal or complex supraspinal analgesic mechanism.

**Trial registration:**

Registered with the Australian New Zealand Clinical Trials Registry (ACTRN12614000682640).

**Electronic supplementary material:**

The online version of this article (doi:10.1186/s12998-016-0128-5) contains supplementary material, which is available to authorized users.

## Background

Spinal manipulative therapy (SMT) is a manual therapy technique used by various health care professions including chiropractors, osteopaths and physiotherapists [1]. Evidence is mixed but some studies suggest that SMT may be effective in managing non-specific spinal pain and some types of headache [2–7]. Since musculoskeletal conditions, particularly low back pain (LBP), represent a significant economic burden and affect a substantial proportion of the population [8, 9], improving our management of these conditions is important.

There is a lack of evidence to explain how SMT may achieve positive clinical outcomes such as pain relief. Improving our understanding of the neurophysiological effects of SMT may improve its clinical use and allow practitioners to make better choices about when and where to apply SMT.

Pressure pain threshold (PPT) is a widely used form of experimental pain, which represents the amount of mechanical pressure required to elicit a nociceptive response at the testing site. It is thought deep Aδ and C sensory fibres are activated at the PPT [10–12].

Pinprick sensitivity (PPS), on the other hand, is not widely used in manual therapy research, and is measured by the self-reported pain response to a sharp stimulus to the skin. PPS is a superficial nociceptive response mediated by Aδ fibres [10, 12].

The short-term effect of SMT on various forms of experimental pain has been studied previously, but many gaps remain. Two recent systematic reviews concluded that SMT has an overall effect of increasing PPT (reducing sensitivity) at sites local to the SMT and remotely [13, 14]. The remote effect may be regional [15, 16] (at a peripheral site innervated by the target spinal region) or systemic [17–20]. SMT also appears to reduce sensitivity to other types of experimental pain [13, 14]. Curiously, PPT increases after cervical SMT but this change has not been shown after lumbar SMT [21–26]. The reason for this is unknown and seems biologically questionable. It could reflect widely differing methods that may have affected the outcomes. The effect of SMT on PPS is unknown. The literature on this topic was considered sufficiently weak to justify further investigation.

The duration of change to pain sensitivity following SMT is also unknown as, in the few studies that have collected data beyond 10 min, findings are mixed [15, 16, 27, 28]. Reductions in pain sensitivity appear to be bilateral [29–33], but there may be asymmetry related to dominant side [31, 32] or side of SMT [29, 30, 33].

### Aims

This research aimed to investigate the effects of lumbar SMT on PPT and PPS, locally and remotely, for 30 min following SMT, and the effects of SMT on the unilateral compared to contralateral side of the body. We were interested in determining whether lumbar SMT had a hypoalgesic effect that was selective to certain stimuli, or to certain regions or sides of the body. Our additional aim was to delineate the short-term time course of any change.

## Methods

This study was a single-blind two-arm randomised trial, and was approved by the Human Research Ethics Committee of Murdoch University (permit 2014/141).

### Participants

Asymptomatic participants aged 18–45 years were recruited from the Murdoch University campus (Perth, Western Australia), via in-class announcements and flyers, and from the general public via word of mouth. Participants were excluded if any of the following applied: (a) current chronic pain condition anywhere, (b) current acute or sub-acute LBP, (c) contraindication to lumbar SMT, (d) qualified chiropractor or student in 4^th^ or 5^th^ year of chiropractic university degree (presumed to be more likely to introduce expectancy bias due to prior knowledge of the neurophysiology of SMT), (e) taken pain-relieving medication in the preceding 24 h, (f) had alcohol within the preceding 12 h.

Originally, an inclusion criterion of naivety to spinal manipulation was included. However, significant difficulty with recruitment led us to remove this and instead add exclusion criterion (d), above.

### Outcome measures

#### Pressure pain threshold

PPT, as a measure of deep mechanical pain sensitivity, was measured using an algometer (FDIX, Wagner Instruments, USA) with a 1 cm^2^ rubber probe. The algometer was validated and standardised against a Kistler Force Plate prior to use in this study (Pearson’s r = 0.99, *p* = .01). For measurement, the algometer was placed perpendicular to the skin and pressure was increased at a rate of 500 g/cm^2^ per second, monitored real-time on the digital algometer display by the assessor. The participant was asked to say “Yes” when the sensation of pressure first changed to pain, at which point the algometer was removed and the maximum pressure recorded. Single measurements at each site were taken following a standard pattern, repeated three times to obtain three measurements per site. This validated approach allowed sufficient rest time between measures at each site [34, 35]. We used a cut-off point of 10 kg/cm^2^ for the forehead and scapula [36], and 12.5 kg/cm^2^ for the lumbar and gastrocnemius sites as this was the upper limit of the algometer. If the cut-off was reached, the cut-off value was used as the measurement for that site and the algometer removed. The average of the second and third measures was used for analysis [36]. An increase in PPT represents a decrease in sensitivity.

#### Pinprick sensitivity

PPS, as a measure of superficial pain sensitivity, was measured using the Neuropen with Neurotips (Owen Mumford, UK). This device is designed to consistently exert 40 g of force when pressed into the skin, though no reliability studies were found for its use. An 11-point Numerical Rating Scale (NRS) was used where 0 = not sharp, and 10 = extremely sharp, as used previously in an experimental trial [37]. The device was placed perpendicular to the skin and pressed in until the guiding markers were aligned; this was maintained for one second and then removed. The participant was then asked to verbally report the intensity of the sharpness using the NRS. Measurements were performed once at each site, immediately following the completion of PPT measures. A new tip was used for each participant. A decrease in PPS represents a decrease in sensitivity.

### Interventions

**Fig. 1:**
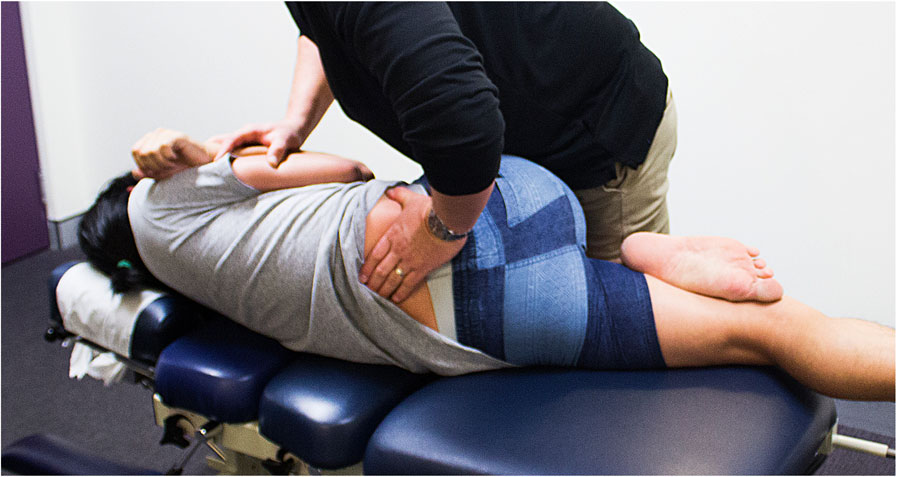
Right L5-S1 SMT technique

A high-velocity low-amplitude SMT was targeted at the L5-S1 spinal segment. A commonly used SMT technique, referred to as the hypothenar mammillary push [38], was used (Fig. 1). The participant was placed in a side-lying position, with the upper leg bent and the lower leg straight. The researcher stabilised the participant at the shoulder with their cephalad hand, and at the thigh with their own leg. A manual contact with the researcher’s caudal hand was then taken over the L5 mamillary process on the right or left side (allocated randomly), the joint was taken to pre-tension, and a high-velocity low-amplitude thrust delivered targeting the L5-S1 facet joint in a posterior to anterior direction. If the clinician thought that the first SMT was ‘unsuccessful’, he was allowed to perform a second SMT. The absence of a facet joint cavitation (audible release) during the SMT was not considered sufficient alone to attempt a second SMT, as there is no evidence that a cavitation is a necessary component of a ‘successful’ manipulation [39–41].

### Procedure

Participants attended a single session at Murdoch University campus, where they completed intake forms, read an information letter, and completed informed consent. Individual participants were given two practice attempts for each outcome measure, applied to the hand, to familiarise them with the procedure. The following four locations were then marked bilaterally on the skin with a non-permanent marker: (a) infraspinatus muscle belly, 2 cm lateral and interior to the root of the spine of the scapula, (b) 2 cm lateral to the L5 spinous process over the paraspinal muscles, (c) mid-portion of the medial gastrocnemius muscle belly, (d) frontal eminence of the forehead. Baseline outcome measures were taken, PPT being measured before PPS, and the assessor then left the room to remain blind to which side SMT was applied. The researcher performing the intervention, a registered chiropractor with 15 year’s clinical and academic experience, randomised participants into one of two groups using the GraphPad random number generator [42] to generate a 1:1 list of 1’s and 2’s, which were placed into sequentially numbered, sealed, opaque envelopes. The envelope was opened immediately prior to the intervention based on order of enrolment into the study. After the intervention was administered, the assessor re-entered the room and measured outcomes immediately, at 10, 20, and 30 min.

### Power analysis

A power analysis using G*Power 3.1 software (University of Düsseldorf, Germany) showed that a sample size of 34 would provide 80 % power for detecting a large effect size of 0.4.

### Data analysis

Data were analysed using SPSS Version 23. A repeated-measures analysis of variance was conducted for PPT and PPS at each location, using factors of time (baseline, immediate, 10, 20, and 30 min), side (side of measurement), and group (side of manipulation, where right SMT = R-SMT and left SMT = L-SMT). Simple contrasts between baseline and each subsequent time point were included in the analyses. Further interactions were investigated using paired t-tests. Effect sizes are reported as partial Eta squared (ƞ_P_^2^), where ≥0.10, ≥0.25, and ≥0.50 are considered to represent small, moderate, and large effect sizes respectively [43].

## Results

**Fig. 2:**
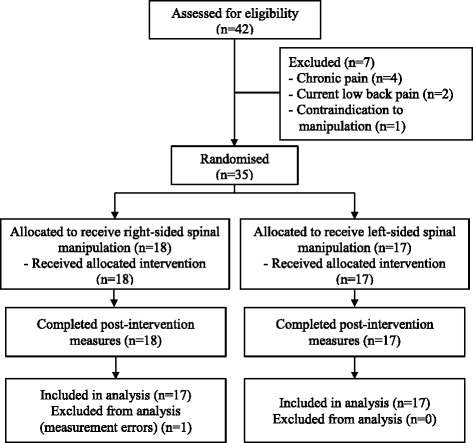
Data collection flow chart

Thirty-four participants (20 male) were recruited for data collection and included in analysis (Fig. 2), with a mean age of 22.6 years (±4.0, range 18–36). Data were collected between October 2014 and June 2015, ending when 34 participants with usable data completed the trial. Baseline characteristics, including PPT and PPS values, are reported in Table 1. No harms were reported during or after follow-up. The intervention was considered successful in all cases at the first attempt; thus, no participant received a second SMT.

**Table 1:** Baseline characteristics

	R-SMT	L-SMT	Actual difference (% difference)
Gender	8 female, 9 male	6 female, 11 male	-
Mean age, years	22.6 (3.1)	22.5 (4.8)	0.1 (0.3)
Dominant hand	11 right, 6 left	16 right, 1 left	-
Calf PPT	5.1 (2.2)	4.5 (1.9)	0.6 (12.2)
Lumbar Spine PPT	7.1 (2.9)	5.9 (2.6)	1.2 (17.3)
Scapula PPT	5.1 (1.9)	4.1 (1.8)	0.9 (18.2)
Forehead PPT	2.8 (1.0)	2.4 (1.0)	0.5 (15.9)
Calf PPS	4.1 (2.2)	5.1 (2.4)	1.0 (19.5)
Lumbar Spine PPS	4.3 (1.6)	5.3 (2.1)	0.9 (17.9)
Scapula PPS	3.2 (1.7)	4.5 (2.0)	1.3 (28.5)
Forehead PPS	4.4 (1.8)	5.3 (2.4)	0.9 (17.3)

*Abbreviations*: *R-SMT* right spinal manipulative therapy group, *L-SMT* left spinal manipulative therapy group, *PPT* pressure pain threshold, *PPS* pinprick sensitivity

Note: where appropriate, data reported as mean (standard deviation), PPT reported in kg/cm^2^, PPS on 11-point numerical rating scale

### Pressure pain threshold

**Fig. 3:**
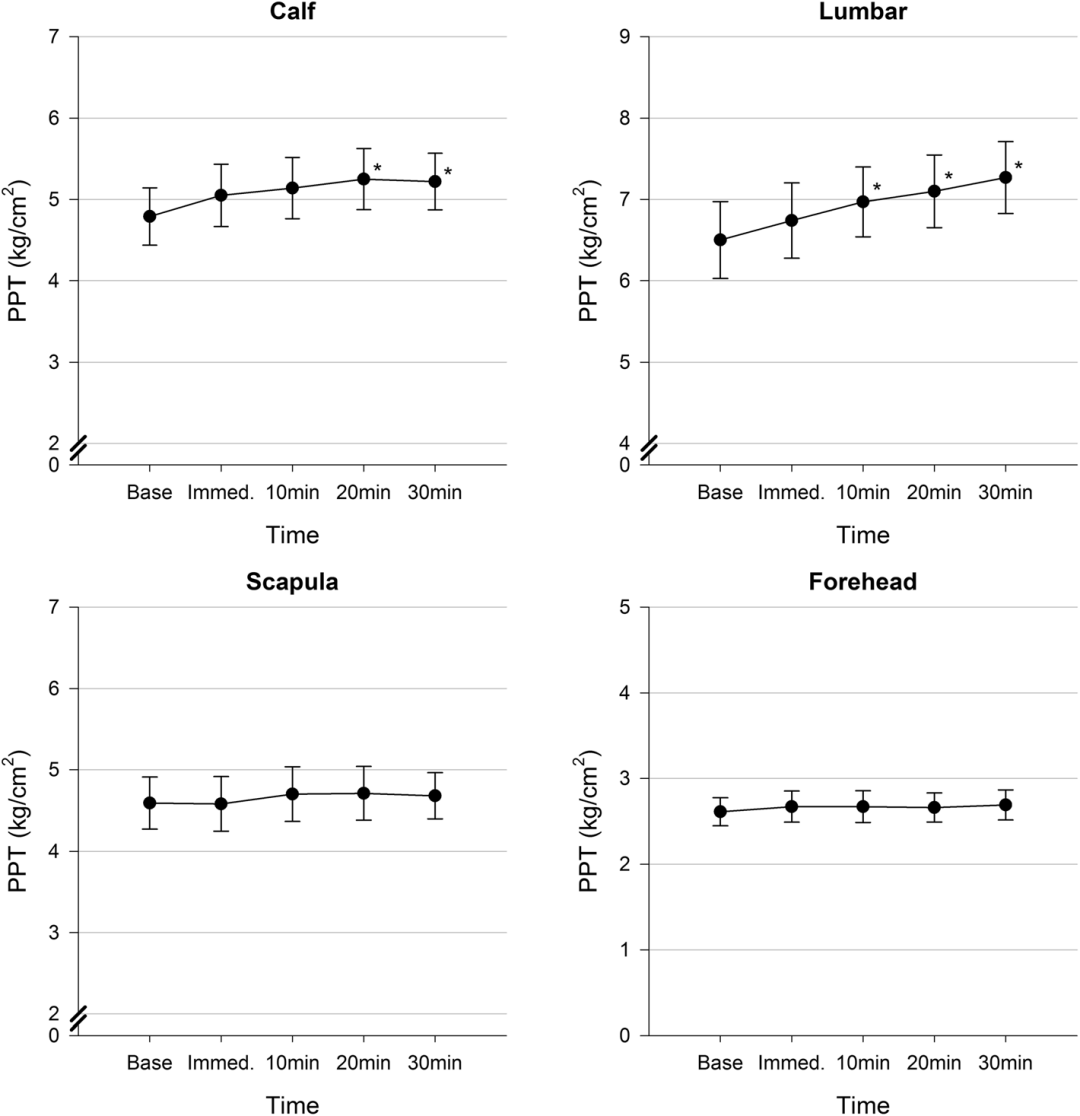
Changes in pressure pain threshold over time with standard error bars. Abbreviations: PPT = pressure pain threshold, Base = baseline, Immed. = immediate, * = p ≤ .05 compared to baseline

Significant effects over time were observed for calf (*p* = .03, ƞ_P_^2^ = .09) and lumbar spine PPT (*p* = .003, ƞ_P_^2^ = .15) with weak effect sizes. Contrasts between baseline and subsequent time points revealed significant increases in PPT from baseline to 20 and 30 min at the calf, and from baseline to 10, 20 and 30 min at the lumbar spine (Table 2, Fig. 3). There was no effect over time for PPT at the scapula (*p* = .71, ƞ_P_^2^ = .01) or forehead (*p* = .67, ƞ_P_^2^ = .01).

**Table 2:** Changes in pressure pain threshold over time

	Mean PPT, kg/cm^2^ (SD)	Difference compared to baseline, kg/cm^2^ (% change)	*p*-value (effect size), compared to baseline
Calf			
Baseline	4.8 (2.1)	-	-
Immediate	5.1 (2.2)	0.3 (5.4 %)	.11 (.08)
10 min	5.1 (2.2)	0.4 (7.3 %)	.05 (.11)
20 min	5.2 (2.2)	0.5 (9.6 %)	.02* (.17)
30 min	5.2 (2.0)	0.4 (9.0 %)	.03* (.14)
Lumbar spine			
Baseline	6.5 (2.8)	-	-
Immediate	6.7 (2.7)	0.2 (3.7 %)	.25 (.04)
10 min	7.0 (2.5)	0.5 (7.2 %)	.03* (.13)
20 min	7.1 (2.7)	0.6 (9.2 %)	.01* (.19)
30 min	7.3 (2.6)	0.8 (11.8 %)	.01* (.21)
Scapula			
Baseline	4.6 (1.9)	-	-
Immediate	4.6 (2.0)	−0.01 (−0.2 %)	.88 (.001)
10 min	4.7 (2.1)	0.1 (2.4 %)	.45 (.02)
20 min	4.7 (1.9)	0.1 (2.6 %)	.44 (.02)
30 min	4.7 (1.7)	0.1 (2.0 %)	.58 (.01)
Forehead			
Baseline	2.6 (1.0)	-	-
Immediate	2.7 (1.1)	0.1 (2.3 %)	.40 (.02)
10 min	2.7 (1.1)	0.1 (2.3 %)	.45 (.02)
20 min	2.7 (1.0)	0.1 (1.9 %)	.58 (.01)
30 min	2.7 (1.0)	0.1 (3.1 %)	.34 (.03)

*Abbreviations*: *PPT* pressure pain threshold, *SD* standard deviation, * = *p* ≤ .05. Note: effect size reported as partial eta squared (ƞ_P_^2^)

Significant effects were observed for side at the calf (*p* = .001, ƞ_P_^2^ = .32) and lumbar spine (*p* = .01, ƞ_P_^2^ = .21) with moderate and weak effect sizes respectively. In particular, PPT was higher on the right compared to the left at both the calf (5.4 kg/cm^2^ and 4.8 kg/cm^2^ respectively) and lumbar spine (7.1 and 6.7 kg/cm^2^ respectively). There was no difference between sides at the scapula (*p* = .87, ƞ_P_^2^ = .001) or forehead (*p* = .64, ƞ_P_^2^ = .01).

No significant differences were detected between groups including across time and side for PPT at the calf, lumbar spine, scapula or forehead.

### Pinprick sensitivity

Significant effects over time were observed for PPS at the calf (*p* = .01, ƞ_P_^2^ = .10), lumbar spine (*p* = .00, ƞ_P_^2^ = .21), and forehead (*p* = .02, ƞ_P_^2^ = .10), each with weak effect sizes, but not at the scapula (*p* = .13, ƞ_P_^2^ = .05). Contrasts revealed significant decreases in PPS at the calf between baseline and 20, and 30 min. At the lumbar spine, significant decreases were noted between baseline and immediate, 10, 20, and 30 min. At the forehead, decreases were noted between baseline and 10, 20, and 30 min. Despite no over-all effect for Time at the scapula, contrasts revealed significant decreases from baseline to 10 min, and 20 min (Table 3).

**Table 3:** Changes in pinprick sensitivity over time

	Mean PPS, 11-point NRS (SD)	Difference compared to baseline, 11-point NRS (% change)	*p*-value (effect size), compared to baseline
Calf			
Baseline	4.6 (2.3)	-	-
Immediate	4.6 (2.1)	−0.02 (−0.4 %)	.94 (.000)
10 min	4.5 (2.4)	−0.1 (−3.2 %)	.51 (.01)
20 min	4.1 (2.2)	−0.5 (−11.5 %)	.04* (.13)
30 min	4.0 (2.2)	−0.6 (−13.6 %)	.02* (.16)
Lumbar Spine			
Baseline	4.8 (1.9)	-	-
Immediate	4.1 (2.1)	−0.6 (−13.4 %)	.003* (.24)
10 min	3.9 (2.3)	−0.9 (−18.0 %)	.001* (.30)
20 min	4.0 (2.0)	−0.8 (−17.1 %)	.001* (.29)
30 min	3.7 (2.2)	−1.1 (−22.5 %)	.000* (.42)
Scapula			
Baseline	3.9 (1.9)	-	-
Immediate	3.6 (2.1)	−0.3 (−6.7 %)	.28 (.04)
10 min	3.4 (1.9)	−0.5 (−12.4 %)	.03* (.15)
20 min	3.5 (2.1)	−0.4 (−9.8 %)	.04* (.13)
30 min	3.5 (2.0)	−0.3 (−8.8 %)	.11 (.08)
Forehead			
Baseline	4.8 (2.1)	-	-
Immediate	4.7 (2.2)	−0.1 (−2.7 %)	.45 (.02)
10 min	4.3 (2.0)	−0.5 (−10.8 %)	.04* (.13)
20 min	4.1 (2.0)	−0.7 (−14.8 %)	.007* (.20)
30 min	4.2 (2.3)	−0.6 (−13.1 %)	.05* (.12)

*Abbreviations*: *PPS* pinprick sensitivity, *NRS* numerical rating scale, *SD* standard deviation, * = *p* ≤ .05. Note: effect size reported as partial eta squared (ƞ_P_^2^)

Significant effects were observed for side at the calf (*p* = .049, ƞ_P_^2^ = .12) and forehead (*p* = .001, ƞ_P_^2^ = .29) with weak and moderate effect sizes respectively. In detail, PPS was found to be higher on the right compared to the left at both the calf (4.5 and 4.3) and forehead (4.6 and 4.2). There was no difference between sides at the lumbar spine (*p* = .91, ƞ_P_^2^ = .00) and scapula (*p* = .63, ƞ_P_^2^ = .01).

A between-group difference was found at the scapula (*p* = .04, ƞ_P_^2^ = .12) with a weak effect size. In detail, the L-SMT group had higher overall scapula PPS compared to the R-SMT group (4.2 and 2.9 respectively). A significant side x group interaction at the scapula was also found (*p* = .03, ƞ_P_^2^ = .14) with weak effect size. The data indicate that in the R-SMT group, right scapula PPS was lower than the left (2.8 and 3.1 respectively), but this was reversed in the L-SMT group (4.4 and 4.0 respectively). These likely reflect baseline differences between groups.

No further significant differences were detected between groups, including across time and side, for PPS at the calf, lumbar spine, scapula or forehead.

### Ipsilateral vs. Contralateral changes

No significant time x side x group changes were detected. However, t-tests between baseline and each subsequent time point indicated that PPT increases were slightly greater on the side ipsilateral to the SMT in the calf particularly, and to a lesser extent in the lumbar spine (see Additional file 1).

## Discussion

This is the first study to observe significant increases in PPT at the lumbar spine and calf following lumbar high-velocity low-amplitude SMT, and to discover that these changes appeared to develop over 10 – 20 min and persist to 30 min.

PPS, an unvalidated outcome measure, was seen to decrease over time at all locations. This could be a systemic real effect or may represent a non-specific effect such as a learned response or adjustment to repeated measurement. Both types of pain sensitivity are mediated by Aδ fibres, but PPT additionally involves C fibres. Since changes in PPT were not systemic in the present study, a systemic treatment effect on PPS is considered unlikely. Further research may be warranted to clarify this.

The magnitude of change in PPT was small, ranging from 7.2 to 11.8 %. The minimum detectable change for PPT has not been clearly defined, but is likely between 35 and 50 % [35, 44, 45]. Percentage change is likely to be most relevant when considering PPT, as absolute baseline values differ widely between testing sites [45]. Based on these minimum detectable change values, our changes may be due to measurement error or chance. The magnitude of changes (as percentage) observed in the present study are similar to some [28] but smaller than other studies [19, 46], but falls within the range identified in the systematic review by Millan et al. [13] of 4.8 to 44.2 %. In our study, as increases in PPT at the calf and lumbar spine were gradual and consistent, and absent at the scapula and forehead, we believe that lumbar SMT evoked a real but small change in PPT.

We observed some asymmetry between the right and left sides. The right calf and lumbar spine were less sensitive overall than the left when measuring PPT, while the right calf and forehead were more sensitive overall than the left when measuring PPS. This is probably a reflection of baseline differences. This finding is at odds with the literature as others have noted no systematic differences in PPT between sides of the body or depending on hand dominance [34, 47, 48]. Our observations may relate to methodological decisions (the left side was always measured before the right), or another unknown factor.

Other literature investigating the effect of SMT on PPT is conflicting. Five studies investigating the effect of lumbar SMT on PPT have found no significant change [21, 23–26]. Four of these measured PPT only immediately following SMT [21, 23–25], so it is possible they may have missed an effect that developed over time, as occurred in our study. The remaining study found a trend toward increasing PPT but this did not reach significance at 30 min [26]. Another study noted, unusually, a significant decrease in PPT after 10 and 15 min [22]. This study did not have a comparison group, and since a nerve conduction study was performed immediately prior to PPT, PPT measures may have been confounded.

The results of studies in the lumbar spine are in stark contrast to cervical spine literature, which quite consistently demonstrate increases in PPT [16, 19, 28–31, 46, 49], and are in agreement with our own study of the lumbar spine. Additionally, other studies have demonstrated mechanical hypoalgesia following lumbar mobilisation [50, 51], lending further strength to our findings. The differences in the literature may relate to methodological differences in terms of PPT testing sites and study populations. Alternatively, differences in mechanoreceptor and nociceptor density, in baseline PPT values, or in the neurophysiologic response to SMT between different spinal regions may explain the inconsistency in the literature [22].

The duration of change in PPT after SMT is not well studied. Cervical and thoracic spine studies have observed increases in PPT at 10 min [19, 20], 15 min [15], 20 min [28], and 30 min [27]. Others have seen no change at 10 min [52], and 2 h [16]. We demonstrated a small change that persisted at 30 min.

Studies that have shown a systemic change over time in PPT following SMT have all failed to show differences when compared to other active treatments or a control condition [17–20], calling into question whether a specific systemic response to SMT occurs. This contrasts with one sham-controlled study [15] and one study comparing two types of SMT which show only local and regional changes following SMT [16]. Our findings suggest that deep mechanical hypoalgesia in response to lumbar SMT is local and regional, but not systemic.

A recent systematic review found no correlation between pain thresholds (including PPT) and subjective pain intensity or disability [53]. PPS does not appear to have been studied in this capacity. It is unknown whether change in PPT following SMT relates to short- or long-term clinical improvement, or if the response differs between healthy and symptomatic populations. Hypothetically, it is possible that a window of hypoalgesia following SMT could promote exercise and physical activity in spinal pain patients. While changes in pain sensitivity following SMT may not currently translate into clinical recommendations, it still represents a promising avenue for experimental research into the neurophysiologic effects of SMT.

Several neurophysiological theories to explain deep mechanical hypoalgesia following SMT have been proposed. These include activation of the descending inhibitory pain control system or activation of the pain gate mechanism [54]. The descending inhibitory pain control system is able to selectively modulate C-fibre nociceptive signals [55], which would be expected to affect PPT (mediated by C- and Aδ-fibres) but not PPS (mediated only by Aδ-fibres). This system is also capable of acting regionally in the spinal cord [56], which could explain the local and regional hypoalgesia we observed. An animal-model experiment demonstrated that mechanical hypoalgesia induced by joint manipulation was mediated by the neurotransmitters serotonin and noradrenaline, both of which are involved in the descending inhibitory pain control system [57]. Thus, activation of the descending inhibitory pain control system may offer a plausible explanation for our findings. The pain gate mechanism is activated only when there is a concurrent non-nociceptive stimulus [58] and thus would likely not account for the more prolonged hypoalgesia we observed. Ultimately, post-SMT hypoalgesia likely arises from a combination of neurophysiologic mechanisms, as well as placebo and psychosocial factors [59].

### Strengths and limitations

There are several strengths to the present study. We followed best practice conditions (based on CONSORT guidelines), and used an a priori power calculation, assessor blinding, participants who were unfamiliar with the neurophysiology of SMT, a single experienced clinician providing interventions, and a validated instrument for measuring PPT.

There are also various limitations. Firstly, we acknowledge that measuring PPS may have had a confounding effect on PPT, though PPS was always measured after PPT and followed by a rest period before the next follow-up to minimise this possibility. PPS was also an unvalidated and subjective measure, and participants’ individual criterion for sharpness intensity may have changed with repeated measurement. Next, anxiety is a known confounder to pain [60], which was not controlled for other than with a thorough informed consent process and assuring participants that SMT was unlikely to cause pain. As our study recruited mainly young, asymptomatic participants, the generalisability of the results is limited when considering older or symptomatic populations. Additionally, we did not have a sham group so some of the effects may be explained by placebo or other non-specific effects such as the positioning or physical touch, or a learned effect. However, it is difficult to envision how such effects would be expressed as regional hypoalgesia. Though participant blinding in manual therapies is difficult [61], a non-thrust manual contact group would have been valuable. Finally, though the study was adequately powered to detect large main effects, it was likely underpowered to detect small changes and two-way and three-way interactions involving side of stimulation or asymmetry of response; thus, we may have committed some type II errors.

## Conclusions

As the only study to date to have demonstrated short-term deep mechanical hypoalgesia following lumbar high-velocity low-amplitude SMT, replication of our results is required before firm conclusions can be drawn. Future research should be directed at measuring mechanical hypoalgesia for at least 30 min following SMT, and comparing the effects of SMT in different spinal regions on pain sensitivity to determine if there are indeed differences between regions. Additionally, furthering our understanding of the neurophysiological pathways that may be involved is important.

## Additional file

Additional file 1: Ipsilateral vs. Contralateral Changes in Pressure Pain Threshold and Pinprick Sensitivity. Description of data: Data tables and figures showing comparisons of change in PPT and PPS on ipsilateral and contralateral sides to SMT. (DOCX 81 kb)

## Abbreviations

LBP: Low back pain
L-SMT: Left-sided spinal manipulative therapy
NRS: Numerical rating scale
PPS: Pinprick sensitivity
PPT: Pressure pain threshold
R-SMT: Right-sided spinal manipulative therapy
SMT: Spinal manipulative therapy
