# Supplementary material for: Effect of lumbar spinal manipulation on local and remote pressure pain threshold and pinprick sensitivity in asymptomatic individuals: a randomised trial
Source: Chiropr Man Therap. 2016 Dec 5;24:47. doi: 10.1186/s12998-016-0128-5 (PMC5137207; doi:10.1186/s12998-016-0128-5)
Supplement: Additional file 1: — Ipsilateral vs. Contralateral Changes in Pressure Pain Threshold and Pinprick Sensitivity. Description of data: Data tables and figures showing comparisons of change in PPT and PPS on ipsilateral and contralateral sides to SMT. (DOCX 81 kb) [file 12998_2016_128_MOESM1_ESM.docx]

**Additional File 1 – Ipsilateral vs. Contralateral Changes in Pressure Pain Threshold and Pinprick Sensitivity**

## Pressure Pain Threshold

Table S1. Paired t-test results for calf PPT in each group on the ipsilateral and contralateral sides, comparing baseline to each follow-up.

|  | ***p*-value (PPT mean difference, kg/cm^2^)** | | | |
| --- | --- | --- | --- | --- |
|  | **R-SMT** | | **L-SMT** | |
|  | **Ipsilateral, right calf** | **Contralateral, left calf** | **Ipsilateral, left calf** | **Contralateral, right calf** |
| **Baseline vs. Immediate** | .09 (0.34) | .51 (0.16) | .05* (0.38) | .66 (0.16) |
| **Baseline vs. 10min** | .11 (0.45) | .98 (0.01) | .01* (0.57) | .15 (0.37) |
| **Baseline vs. 20min** | .12 (0.48) | .58 (0.16) | .01* (0.76) | .20 (0.42) |
| **Baseline vs. 30min** | .18 (0.39) | .99 (0.00) | .01* (0.71) | .06 (0.60) |

Abbreviations: PPT = pressure pain threshold, R-SMT = right-sided spinal manipulative therapy, L-SMT = left-sided spinal manipulative therapy, * = p ≤ .05.

Figure S1. Mean calf PPT after SMT in each group on the ipsilateral and contralateral sides, comparing baseline to each follow-up with standard error bars.

Abbreviations: PPT = pressure pain threshold, R-SMT = right-sided spinal manipulative therapy, L-SMT = left-sided spinal manipulative therapy, Ipsilat. = ipsilateral, Contralat. = contralateral, * = p ≤ 0.05 compared to baseline.

Table S2. Paired t-test results for lumbar spine PPT in each group on the ipsilateral and contralateral sides, comparing baseline to each follow-up.

|  | ***p*-value (PPT mean difference, kg/cm^2^)** | | | |
| --- | --- | --- | --- | --- |
|  | **R-SMT** | | **L-SMT** | |
|  | **Ipsilateral, right lumbar spine** | **Contralateral, left lumbar spine** | **Ipsilateral, left lumbar spine** | **Contralateral, right lumbar spine** |
| **Baseline vs. Immediate** | .11 (0.55) | .71 (-0.14) | .38 (0.30) | .44 (0.21) |
| **Baseline vs. 10min** | .14 (0.67) | .62 (0.14) | .07 (0.61) | .18 (0.47) |
| **Baseline vs. 20min** | .04* (0.92) | .34 (0.39) | .12 (0.48) | .01* (0.60) |
| **Baseline vs. 30min** | .05* (0.99) | .31 (0.50) | .03* (0.69) | .02* (0.90) |

Abbreviations: PPT = pressure pain threshold, R-SMT = right-sided spinal manipulative therapy, L-SMT = left-sided spinal manipulative therapy, * = p ≤ .05.

Figure S2. Mean lumbar spine PPT after SMT in each group on the ipsilateral and contralateral sides, comparing baseline to each follow-up with standard error bars.

Abbreviations: PPT = pressure pain threshold, R-SMT = right-sided spinal manipulative therapy, L-SMT = left-sided spinal manipulative therapy, Ipsilat. = ipsilateral, Contralat. = contralateral, * = p ≤ 0.05 compared to baseline.

Table S3. Paired t-test results for scapula PPT in each group on the ipsilateral and contralateral sides, comparing baseline to each follow-up.

|  | ***p*-value (PPT mean difference, kg/cm^2^)** | | | |
| --- | --- | --- | --- | --- |
|  | **R-SMT** | | **L-SMT** | |
|  | **Ipsilateral, right scapula** | **Contralateral, left scapula** | **Ipsilateral, left scapula** | **Contralateral, right scapula** |
| **Baseline vs. Immediate** | .62 (0.09) | .19 (0.18) | .79 (0.05) | .08 (-0.38) |
| **Baseline vs. 10min** | .31 (0.31) | .23 (0.31) | .53 (-0.10) | .38 (-0.10) |
| **Baseline vs. 20min** | .51 (0.19) | .82 (-0.06) | .04* (0.31) | .98 (-0.00) |
| **Baseline vs. 30min** | .97 (0.01) | .40 (0.24) | .74 (0.07) | .93 (0.02) |

Abbreviations: PPT = pressure pain threshold, R-SMT = right-sided spinal manipulative therapy, L-SMT = left-sided spinal manipulative therapy, * = p ≤ .05.

Figure S3. Mean scapula PPT after SMT in each group on the ipsilateral and contralateral sides, comparing baseline to each follow-up with standard error bars.

Abbreviations: PPT = pressure pain threshold, R-SMT = right-sided spinal manipulative therapy, L-SMT = left-sided spinal manipulative therapy, Ipsilat. = ipsilateral, Contralat. = contralateral, * = p ≤ 0.05 compared to baseline.

Table S4. Paired t-test results for forehead PPT in each group on the ipsilateral and contralateral sides, comparing baseline to each follow-up.

|  | ***p*-value (PPT mean difference, kg/cm^2^)** | | | |
| --- | --- | --- | --- | --- |
|  | **R-SMT** | | **L-SMT** | |
|  | **Ipsilateral, right forehead** | **Contralateral, left forehead** | **Ipsilateral, left forehead** | **Contralateral, right forehead** |
| **Baseline vs. Immediate** | .34 (-0.09) | .30 (0.10) | .43 (0.09) | .32 (0.15) |
| **Baseline vs. 10min** | .99 (-0.00) | .63 (0.07) | .45 (0.07) | .38 (0.12) |
| **Baseline vs. 20min** | .51 (-0.07) | .33 (0.13) | .46 (0.11) | .80 (0.04) |
| **Baseline vs. 30min** | .80 (0.04) | .55 (0.09) | .40 (0.10) | .37 (0.12) |

Abbreviations: PPT = pressure pain threshold, R-SMT = right-sided spinal manipulative therapy, L-SMT = left-sided spinal manipulative therapy.

## Pinprick Sensitivity

Table S5. Paired t-test results for calf PPS in each group on the ipsilateral and contralateral sides, comparing baseline to each follow-up.

|  | ***p*-value (PPS mean difference, 11-point NRS)** | | | |
| --- | --- | --- | --- | --- |
|  | **R-SMT** | | **L-SMT** | |
|  | **Ipsilateral, right calf** | **Contralateral, left calf** | **Ipsilateral, left calf** | **Contralateral, right calf** |
| **Baseline vs. Immediate** | .55 (0.18) | .87 (0.6) | .99 (0.00) | .52 (-0.29) |
| **Baseline vs. 10min** | .85 (-0.06) | .79 (-0.12) | .87 (-0.06) | .37 (-0.35) |
| **Baseline vs. 20min** | .28 (-0.41) | .08 (-0.71) | .06 (-0.82) | .64 (-0.18) |
| **Baseline vs. 30min** | .39 (-0.29) | .04* (-0.77) | .16 (-0.65) | .12 (-0.82) |

Abbreviations: PPS = pinprick sensitivity, NRS = numerical rating scale, R-SMT = right-sided spinal manipulative therapy, L-SMT = left-sided spinal manipulative therapy, * = p ≤ .05.

Table S6. Paired t-test results for lumbar spine PPS in each group on the ipsilateral and contralateral sides, comparing baseline to each follow-up.

|  | ***p*-value (PPS mean difference, 11-point NRS)** | | | |
| --- | --- | --- | --- | --- |
|  | **R-SMT** | | **L-SMT** | |
|  | **Ipsilateral, right lumbar spine** | **Contralateral, left lumbar spine** | **Ipsilateral, left lumbar spine** | **Contralateral, right lumbar spine** |
| **Baseline vs. Immediate** | .11 (-0.65) | .62 (-0.18) | .02* (-0.82) | .01* (-0.94) |
| **Baseline vs. 10min** | .05* (-0.82) | .02* (-1.06) | .04* (-0.77) | .03* (-0.82) |
| **Baseline vs. 20min** | .06 (-0.88) | .09 (-0.59) | .06 (-0.88) | .004* (-0.94) |
| **Baseline vs. 30min** | .02* (-1.00) | .07 (-0.88) | .003* (-1.29) | .01* (-1.18) |

Abbreviations: PPS = pinprick sensitivity, NRS = numerical rating scale, R-SMT = right-sided spinal manipulative therapy, L-SMT = left-sided spinal manipulative therapy, * = p ≤ .05.

Table S7. Paired t-test results for scapula PPS in each group on the ipsilateral and contralateral sides, comparing baseline to each follow-up.

|  | ***p*-value (PPS mean difference, 11-point NRS)** | | | |
| --- | --- | --- | --- | --- |
|  | **R-SMT** | | **L-SMT** | |
|  | **Ipsilateral, right scapula** | **Contralateral, left scapula** | **Ipsilateral, left scapula** | **Contralateral, right scapula** |
| **Baseline vs. Immediate** | .06 (-0.82) | .64 (0.18) | .78 (-0.12) | .48 (-0.29) |
| **Baseline vs. 10min** | .09 (-0.82) | .74 (-0.12) | .03* (-0.71) | .31 (-0.29) |
| **Baseline vs. 20min** | .04* (-0.65) | .99 (0.00) | .20 (-0.47) | .20 (-0.41) |
| **Baseline vs. 30min** | .12 (-0.71) | .99 (0.00) | .31 (-0.29) | .33 (-0.35) |

Abbreviations: PPS = pinprick sensitivity, NRS = numerical rating scale, R-SMT = right-sided spinal manipulative therapy, L-SMT = left-sided spinal manipulative therapy, * = p ≤ .05.

Table S8. Paired t-test results for forehead PPS in each group on the ipsilateral and contralateral sides, comparing baseline to each follow-up.

|  | ***p*-value (PPS mean difference, 11-point NRS)** | | | |
| --- | --- | --- | --- | --- |
|  | **R-SMT** | | **L-SMT** | |
|  | **Ipsilateral, right forehead** | **Contralateral, left forehead** | **Ipsilateral, left forehead** | **Contralateral, right forehead** |
| **Baseline vs. Immediate** | .61 (-0.12) | .33 (-0.24) | .61 (-0.18) | .99 (0.00) |
| **Baseline vs. 10min** | .13 (-0.53) | .05* (-0.59) | .35 (-0.47) | .23 (-0.47) |
| **Baseline vs. 20min** | .20 (-0.47) | .13 (-0.59) | .04* (-1.12) | .07 (-0.65) |
| **Baseline vs. 30min** | .23 (-0.47) | .02* (-0.94) | .21 (-0.71) | .44 (-0.41) |

Abbreviations: PPS = pinprick sensitivity, NRS = numerical rating scale, R-SMT = right-sided spinal manipulative therapy, L-SMT = left-sided spinal manipulative therapy, * = p ≤ .05.
